# Supplementary figures and images for: Selection and Validation of Reference Genes in Different Tissues of Okra (Abelmoschus esculentus L.) under Different Abiotic Stresses
Source: Genes (Basel). 2023 Feb 27;14(3):603. doi: 10.3390/genes14030603 (PMC10048105; doi:10.3390/genes14030603)

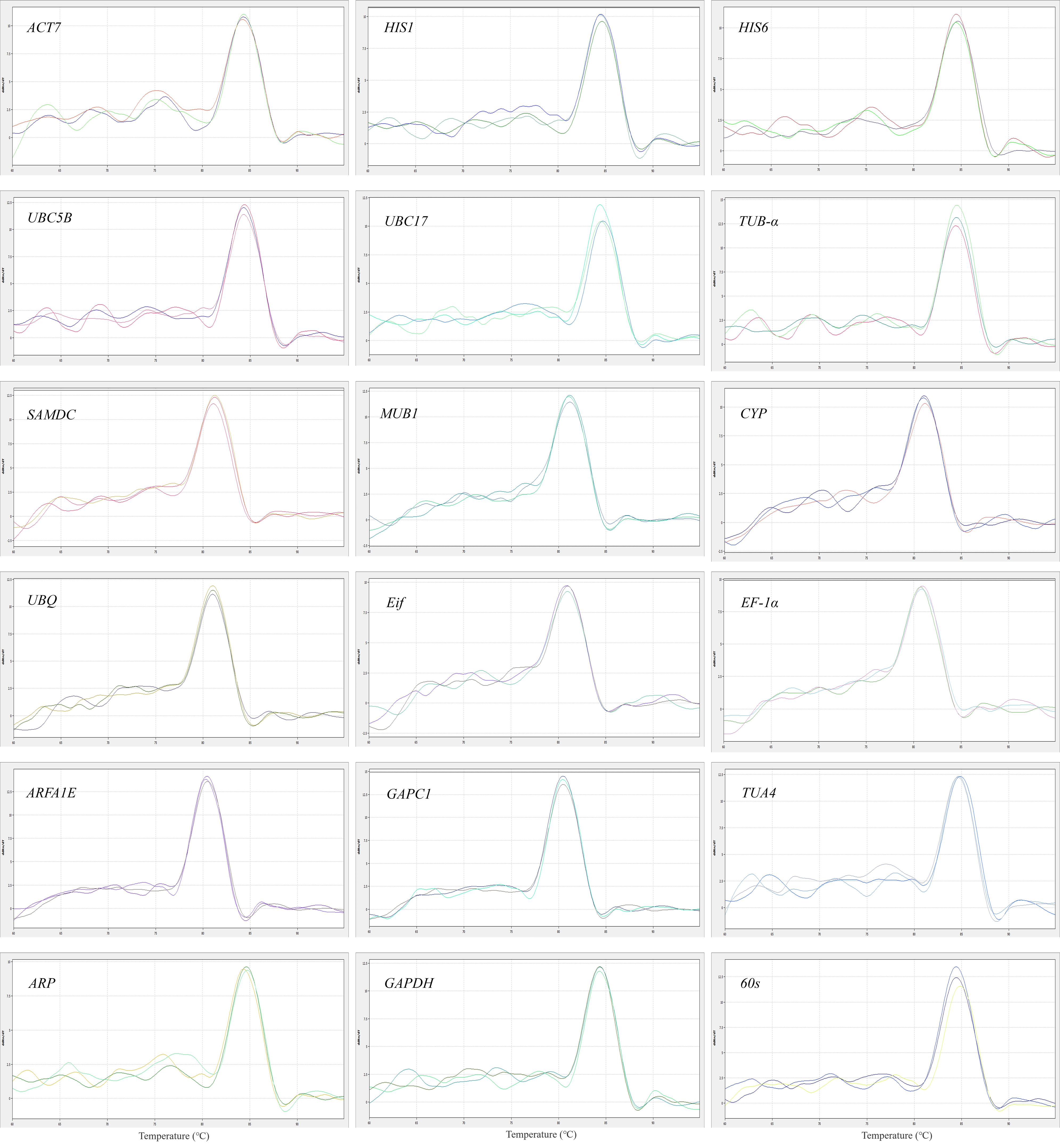

Supplement: Supplementary file 1 [file genes-14-00603-s001.zip › Figure S1.tif]

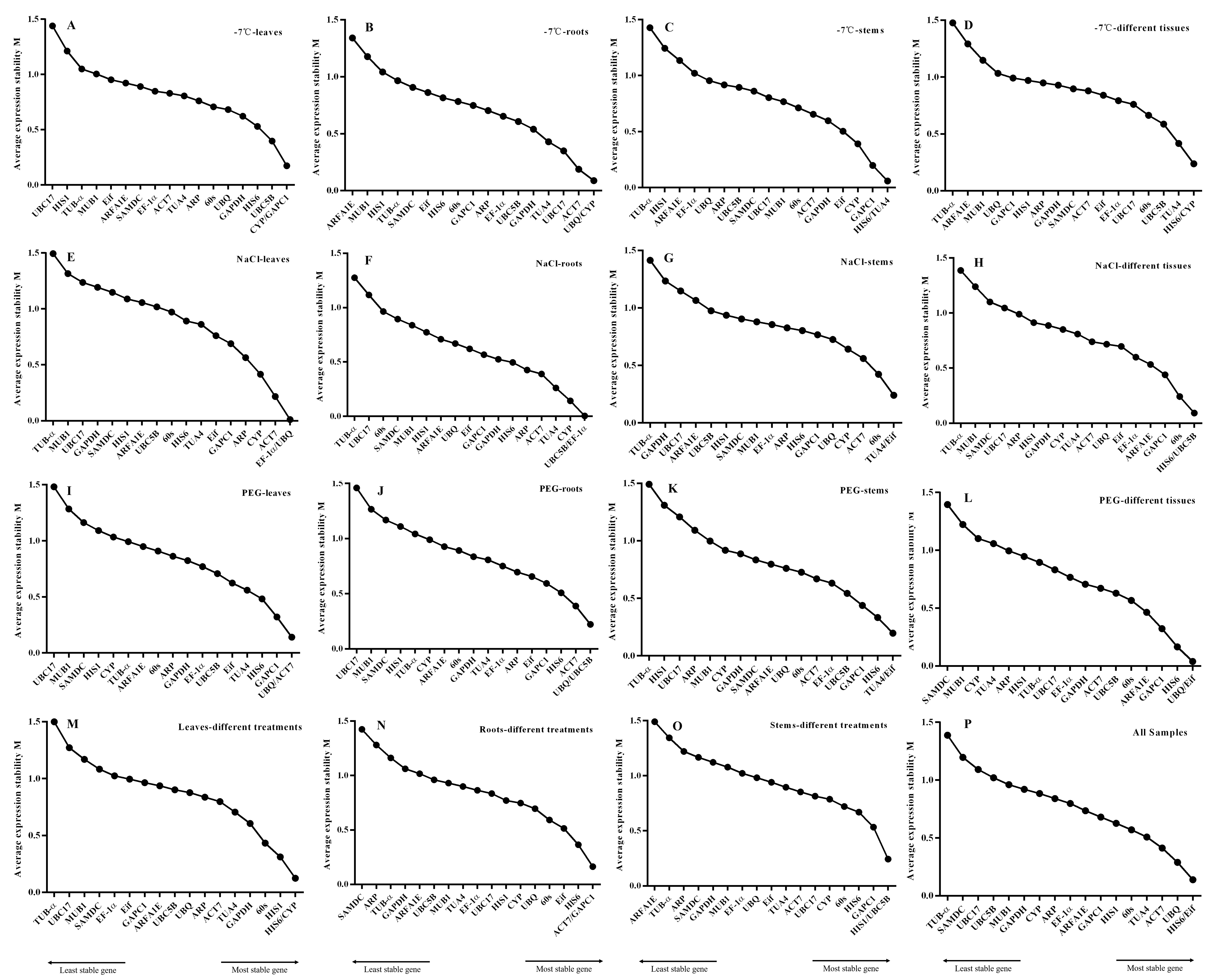

Supplement: Supplementary file 1 [file genes-14-00603-s001.zip › Figure S2.tif]
